# Neuroimaging findings in newborns with congenital heart disease prior to surgery: an observational study

**DOI:** 10.1136/archdischild-2018-314822

**Published:** 2019-06-26

**Authors:** Christopher J Kelly, Sophie Arulkumaran, Catarina Tristão Pereira, Lucilio Cordero-Grande, Emer J Hughes, Rui Pedro A G Teixeira, Johannes K Steinweg, Suresh Victor, Kuberan Pushparajah, Joseph V Hajnal, John Simpson, A David Edwards, Mary A Rutherford, Serena J Counsell

**Affiliations:** 1 Centre for the Developing Brain, School of Imaging Sciences and Biomedical Engineering, King’s College London, London, UK; 2 School of Imaging Sciences and Biomedical Engineering, King’s College London, London, UK; 3 Paediatric Cardiology Department, Evelina London Children’s Healthcare, London, UK; 4 Congenital Heart Disease, Evelina London Children’s Hospital, London, London, UK

**Keywords:** neonatology, cardiology, neurodevelopment, imaging, cardiac surgery

## Abstract

**Objectives:**

Neurodevelopmental impairment has become the most important comorbidity in infants with congenital heart disease (CHD). We aimed to (1) investigate the burden of brain lesions in infants with CHD prior to surgery and (2) explore clinical factors associated with injury.

**Study design:**

Prospective observational study.

**Setting:**

Single centre UK tertiary neonatal intensive care unit.

**Patients:**

70 newborn infants with critical or serious CHD underwent brain MRI prior to surgery.

**Main outcome measures:**

Prevalence of cerebral injury including arterial ischaemic strokes (AIS), white matter injury (WMI) and intracranial haemorrhage.

**Results:**

Brain lesions were observed in 39% of subjects (95% CI 28% to 50%). WMI was identified in 33% (95% CI 23% to 45%), subdural haemorrhage without mass effect in 33% (95% CI 23% to 45%), cerebellar haemorrhage in 9% (95% CI 4% to 18%) and AIS in 4% (95% CI 1.5% to 12%). WMI was distributed widely throughout the brain, particularly involving the frontal white matter, optic radiations and corona radiata. WMI exhibited restricted diffusion in 48% of cases. AIS was only observed in infants with transposition of the great arteries (TGA) who had previously undergone balloon atrial septostomy (BAS). AIS was identified in 23% (95% CI 8% to 50%) of infants with TGA who underwent BAS, compared with 0% (95% CI 0% to 20%) who did not.

**Conclusions:**

Cerebral injury in newborns with CHD prior to surgery is common.

**What is already known on this topic?**

- Neurodevelopmental impairment is a common comorbidity in major congenital heart disease (CHD), affecting up to half of children.
- Prior imaging studies have identified brain lesions in newborn infants prior to surgery, with a prevalence that varies between 19% and 52% of cases.
- Abnormal brain development in CHD is observed in utero, with a derailment of normal growth becoming most pronounced in the third trimester.

**What this study adds?**

- Brain lesions were identified in 39% (95% CI 28% to 50%) of preoperative infants with major CHD, in a contemporary UK cohort.
- White matter injury was the most common finding, with a widespread distribution throughout the brain.

## Introduction

Congenital heart disease (CHD) is the most common congenital defect,^1^ affecting ~1% of newborns.^2^ Historically, few infants born with major CHD survived to adulthood,^3^ but advances in diagnostic, interventional and surgical techniques over recent decades have led to dramatic increases in survival.^4^ However, children with CHD remain at risk of neurodevelopmental impairment, characterised by mild cognitive impairment, impaired social and communication skills, inattention and later, deficits in executive function.^5–12^

The aetiology of neurodevelopmental impairment in CHD remains poorly understood. It has become clear that neurological insult in CHD begins before surgery, with altered neurological state in the newborn period,^13^ population studies demonstrating reduced birth weight and head circumference,^14^ and in utero imaging studies showing a derailed trajectory of brain growth in the third trimester.^15 16^

Studies of brain injury in presurgical newborns have reported lesions including white matter injury (WMI) and stroke with a prevalence that varies considerably from 19% to 52% of cases.^17^ The cause for such variation between cohorts remains unclear, and may be due to differences in local practice, differences in cohort representation of each CHD diagnosis, or variability in reporting definitions. This study aimed to (1) characterise brain lesions using MRI in a contemporary UK cohort of newborns with major CHD prior to surgery and (2) assess which clinical factors are associated with brain injury.

## Methods

### Study design and participants

We recruited a prospective cohort of 70 infants with serious or critical CHD, born September 2014 to November 2017, from the Neonatal Intensive Care Unit at St Thomas’ Hospital, London. Following a previously published UK categorisation,^18 19^ ‘critical’ CHD was defined as infants with hypoplastic left heart syndrome (HLHS), pulmonary atresia with intact ventricular septum, simple transposition of the great arteries (TGA), interruption of the aortic arch and all infants dying or requiring surgery within the first 28 days of life with the following conditions: coarctation of the aorta (CoA); aortic valve stenosis; pulmonary valve stenosis; tetralogy of Fallot (TOF); pulmonary atresia with ventricular septal defect (VSD); total anomalous pulmonary venous connection. ‘Serious’ CHD was defined as any cardiac lesion not defined as critical, which requires intervention (cardiac catheterisation or surgery) or results in death between 1 month and 1 year of age. Infants were excluded if they had clinical evidence of a congenital syndrome or malformation, a suspected or confirmed major chromosomal abnormality (eg, aneuploidy), any previous neonatal surgery or who had a suspected congenital infection. Informed written parental consent was obtained.

### MRI

MRI was performed on a 3-Tesla system (Philips Achieva, Best, Netherlands), situated on the Neonatal Intensive Care Unit at St Thomas’ Hospital, as soon as the infant could be safely transferred to the scanner and before undergoing surgery. All examinations were supervised by an experienced paediatrician, and scanned in natural sleep without sedation, as described previously.^20^ MRI sequences are described in table 1, and included a 5 s initial slow ramp-up in acoustic noise to avoid eliciting a startle response. T1-weighted and T2-weighted images were motion-corrected post hoc using a dedicated motion-corrected reconstruction.^21–23^

**Table 1:** MRI sequence parameters

Sequence	Repetitiontime (TR) (ms)	Echo time (TE) (ms)	Flip angle	Acquired voxel size (mm)	Reconstructed voxel size (mm)	Other parameters
September 2014 to November 2015 (n=18, 26%), adult 32-channel head coil						
T1-weighted (magnetisation prepared rapid acquisition gradient echo)	17	4.6	13°	0.82×0.97×1	0.82×0.82×0.5	TI: 1465 ms
T2-weighted (fast-spin echo)	14 473	160	90°	1.15×1.18×2	0.86×0.86×2	–
Diffusion-weighted imaging	7536	49	90°	2×2×2	1.75×1.75×2	32 directions, b=0, 750 s/mm^2^
Susceptibility-weighted imaging (spoiled gradient-recalled echo)	32	25	12°	0.45×0.45×1.8	0.4×0.4×1.8	–
Venogram	18	6.7	10°	0.9×0.9×2	0.44×0.44×1	Phase contrast velocity 15 cm/s
November 2015 to November 2017 (n=52, 74%), neonatal 32-channel head coil and positioning device^20^						
T1-weighted (magnetisation prepared rapid acquisition gradient echo)	11	4.6	9°	0.81×0.8×0.8	0.76×0.76×0.8	TI: 714 ms
T2-weighted multislice turbo spin echo, sagittal and axial, combined in reconstruction	12	156	90°	0.81×0.82×1.6	0.8×0.8×0.8	–
Diffusion-weighted imaging^72^	3800	90	90°	1.5×1.5×3, slice gap −1.5 mm	1.17×1.17×3, slice gap −1.5 mm	300 directions, b=0, 400, 1000, 2600
Susceptibility-weighted imaging (spoiled gradient-recalled echo)	32	25	12°	0.45×0.45×1.8	0.4×0.4×1.8	–
Venogram	18	6.7	10°	0.9×0.9×2	0.44×0.44×1	Phase contrast velocity 15 cm/s

### Image review

Images were reported by two neonatal neuroradiologists. All images were subsequently rereviewed to ensure consistency, and lesions classified as focal arterial ischaemic stroke (AIS), WMI, cerebellar haemorrhage or intraventricular haemorrhage. The location and properties of lesions on T1-weighted and T2-weighted imaging, susceptibility-weighted imaging (SWI) and apparent diffusion coefficient (ADC) map were recorded. AIS was defined as a homogeneous single area of altered signal occurring within the territory of a major cerebral artery, involving the cerebral cortical or deep nuclear grey matter, with restricted diffusion demonstrated as low signal on the ADC map, and/or hyperintensity on T2-weighted images.^24^ Additionally, following a previously published classification,^25^ WMI was classified into normal (no WMI), mild (≤3 foci and all ≤2 mm), moderate (>3 and ≤10 foci or any >2 mm) or severe (>10 foci). Subdural haemorrhage was recorded but was not considered brain injury, given its frequent occurrence in the healthy neonatal population.

### Generation of WMI maps

White matter lesions were segmented by a single reader (CTP) from T1-weighted images using ITK-SNAP,^26^ and confirmed by consensus of three authors (SC, CK, CTP). WMI was defined as discrete areas of high signal intensity on T1-weighted imaging, usually accompanied by low signal intensity on T2-weighted imaging.^27–29^ T2-weighted and T1-weighted images for each subject were coregistered using a rigid body registration (FLIRT).^30^ A group template was then created from both modalities using symmetric diffeomorphic normalisation for multivariate neuroanatomy and a cross-correlation similarity metric using advanced normalization tools (ANTs).^31^ T1-weighted images were non-linearly registered to the template using symmetric diffeomorphic normalisation. These registrations were then used to transform individual lesion maps into template space. Finally, a probabilistic map was calculated from the mean of all lesion maps in template space, visualised using mrtrix3.^32^ Descriptive statistics were generated using MATLAB (2015b, MathWorks, USA). Total brain volume was calculated using a previously validated neonatal-specific segmentation pipeline.^33^

### Statistical analysis

Categorical clinical variables between those with and without injury of interest were compared using Fisher’s exact tests and CIs for the magnitude of the difference were calculated using a two-sample test for equality of proportions. Continuous variables were tested for normality using the Shapiro-Wilk test. For normally distributed continuous variables, we determined means and SD for those with and without injury, and compared groups with the Student’s t-test. For non-normal continuous and ordinal variables, we determined medians and IQR for those with and without injury, and compared groups using the Mann-Whitney U test (p values were not reported for sample sizes <5), with CIs calculated using Wilson’s methods. Analyses were performed using SPSS V.24 and R V.3.5.1 (r-project.org).

## Results

### Subject data

We enrolled 70 infants, all of whom were scanned prior to surgery. T1-weighted and T2-weighted images were acquired in 100% of subjects, ADC map in 99% (1 motion corrupted), SWI in 93% (2 motion corrupted, 3 awoke prior to sequence) and MRV in 80% (13 awoke before protocol end, 1 poor quality). The median gestational age (GA) at birth was 38.3 weeks (IQR 37.4–38.7), and at scan was 39.0 weeks (IQR 38.4–39.7). The average age at scan was 5 days (IQR 2–7). The median age at intervention (cardiac catheterisation or surgery) was 13.0 days (IQR 4.0–32.8). Cardiac surgery was performed at a median of 10.0 days (IQR 6.0–40.0) following the scan (online supplementary figure 1). Clinical variables are presented in table 2. No patients experienced birth asphyxia. Antenatal diagnosis of CHD was made in 97% (n=68), of whom all were born at our institution. Both postnatally diagnosed cases were outborn.

10.1136/archdischild-2018-314822.supp1 Supplementary data

**Table 2:** Clinical characteristics of the cohort. Data are n (%) or median (IQR), unless otherwise specified. Percentages are column-wise for totals and row-wise for subgroups

Variable	Total (n=70)
Sex	
Female	33 (47%)
Male	37 (53%)
Delivery method	
Normal vaginal delivery	27 (39%)
Forceps vaginal delivery	6 (9%)
Ventouse vaginal delivery	6 (9%)
Emergency caesarean	19 (27%)
Elective caesarean	12 (17%)
Induction of labour	41 (59%)
Prenatal diagnosis	68 (97%)
Outborn	2 (3%)
Gestational age at birth (weeks)	38.3 (37.4–38.7)
Gestational age at preoperative MRI (weeks)	39.0 (38.4–39.7)
Age at scan (days)	5 (2–7)
Birth weight (kg) (mean, SD)*	2.94 (0.57)
Head circumference (cm)	33.5 (32.2–34.5)
Apgar score	
1 min	9 (7–9)
5 min	9 (9–10)
Cord arterial pH (mean, SD)*	7.29 (0.082)
Prostaglandin E2	31 (44%)
Cardiac arrest	0 (0%)
Days mechanical ventilation	0 (0–1)
Balloon atrial septostomy	13 (19%)
Umbilical	6 (46%)
Femoral	7 (54%)
Heart lesion	
Transposition of the great arteries	28 (40%)
Tetralogy of Fallot	13 (19%)
Coarctation of the aorta	11 (16%)
Pulmonary atresia	5 (7%)
Hypoplastic left heart syndrome	4 (6%)
Pulmonary stenosis	3 (4%)
Truncus arteriosus	3 (4%)
Tricuspid atresia	2 (3%)
Large VSD	1 (1%)

*Normally distributed variables, summarised by mean and SD.

### Preoperative brain injury in CHD

**Figure 1:**
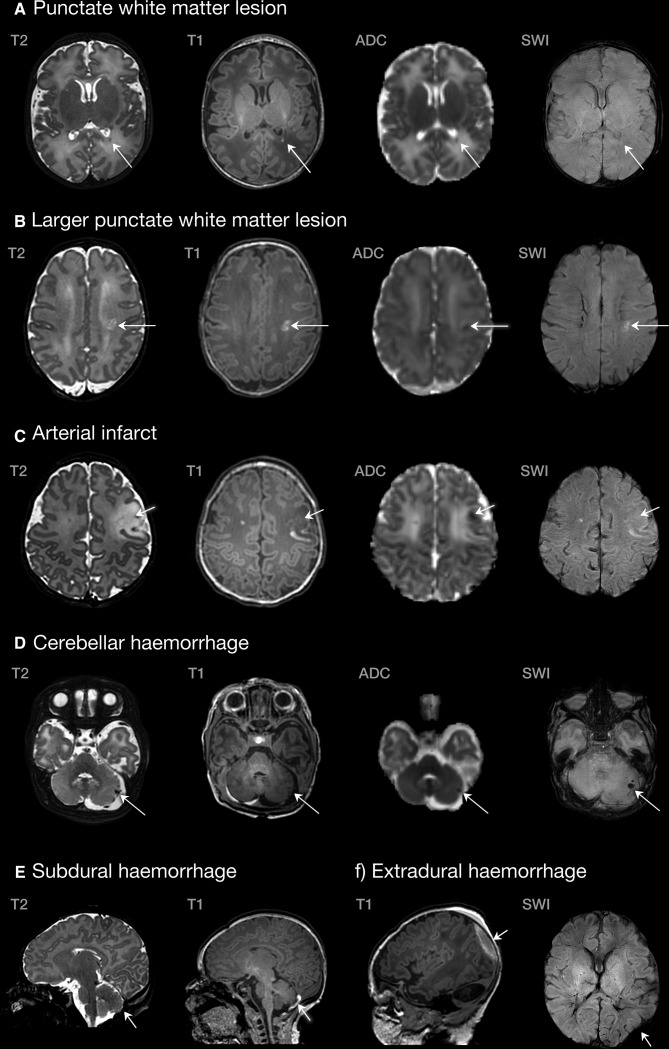
Examples of lesions identified in the congenital heart disease cohort. (A) Single lesion in the posterior periventricular white matter (TGA, scanned at 39+6); (B) larger white matter lesion in the centrum semiovale (pulmonary atresia, scanned at 37+2); (C) left middle cerebral artery infarct (TGA, scanned at 39+5); (D) cerebellar haemorrhage (CoA, scanned at 39+3); (E) subdural haemorrhage (TGA, scanned at 39+2); (F) extradural haemorrhage (CoA, scanned 39+3). ADC, apparent diffusion coefficient; CoA, coarctation of  the  aorta; SWI, susceptibility-weighted imaging; TGA, transposition of the great arteries.

Cerebral lesions were identified in 39% (n=27, 95% CI 28% to 50%) of cases, including WMI in 33% (n=23, 95% CI 23% to 45%; figure 1A, B), cerebellar haemorrhage in 9% (n=6, 95% CI 4% to 18%; figure 1D) and AIS in 4% (n=3, 95% CI 1.5% to 12%; figure 1C). A summary of lesions observed in this cohort is presented in table 3. Mild WMI was observed in 20% (95% CI 12 to 31), moderate WMI in 6% (95% CI 4 to 17) and severe in 1% (95% CI 0.3 to 8) of cases. Forty-three infants (61%, 95% CI 50% to 72%) had no evidence of lesions, while seven (10%, 95% CI 5% to 19%) had more than one lesion type. All cases of AIS were clinically silent, and were located in the left middle cerebral artery territory. There were no cases of sinus venous thrombosis. Asymmetrical transverse sinus flow was noted in 31% of infants (n=22, 95% CI 22% to 43%), with reduced left-sided flow in 91% of these (n=20, 95% CI 72% to 97%), a common anatomical variant.^34^ There were no cases of intraventricular haemorrhage. Other intracranial findings included subdural haemorrhage without mass effect in 33% (n=23, 95% CI 23% to 44.5%, figure 1E), subependymal cysts in 11% (n=8, 95% CI 6% to 21%), cerebellar vermis rotation in 7% (n=5, 95% CI 3% to 16%) and extradural haematoma in 1% (n=1, 95% CI 0.25% to 8%, figure 1F).

**Table 3:** Injury by cardiac physiology group. Numbers in brackets refer to 95% CI. Altered streaming includes infants with TGA and truncus arteriosus. Left-sided lesions includes CoA and HLHS. Right-sided lesions includes TOF, pulmonary atresia, pulmonary stenosis and tricuspid atresia. P value reflects distribution across cardiac physiology groups compared using χ^2^ tests

Brain injury type	Cardiac physiology group								
	Altered streaming		Left-sided lesion		Right-sided lesion		Total		P value
	n	%	n	%	n	%	n	%	
WMI: all	11	32 (19 to 49)	5	36 (16 to 61)	7	32% (16 to 53)	23	33 (23 to 44)	0.967
WMI category									0.734
Mild	5	15 (6 to 30)	3	21 (8 to 48)	6	27 (13 to 48)	14	20 (12 to31)	–
Moderate	3	9 (3 to 23)	2	14 (4 to 40)	1	5 (1 to 22)	6	9 (4 to 17)	–
Severe	1	3 (1 to 15)	0	0 (0 to 22)	0	0 (0 to 15)	1	1 (0.3 to 8)	–
WMI with infarct	2	6 (2 to 19)	0	0 (0 to 22)	0	0 (0 to 15)	2	3 (1 to 10)	–
Arterial ischaemic stroke	3	9 (3 to 23)	0	0 (0 to 22)	0	0 (0 to 15)	3	4 (1 to 12)	0.190
Cerebellar haemorrhage	2	6 (2 to 19)	2	14 (4 to 40)	2	9 (3 to 28)	6	9 (4 to 17)	0.636
Parenchymal haemorrhage	0	0 (0 to 10)	0	0 (0 to 22)	0	0 (0 to 15)	0	0 (0 to 5)	1.000
Venous sinus thrombosis	0	0 (0 to 10)	0	0 (0 to 22)	0	0 (0 to 15)	0	0 (0 to 5)	1.000
Subdural haemorrhage	12	35 (21 to 52)	6	43 (21 to 67)	5	23 (10 to 43)	23	33 (23 to 44)	0.417
Total	34		14		22		70		

CoA,  coarctation of the aorta; HLHS, hypoplastic left heart syndrome; TOF, tetralogy of Fallot; WMI, white matter injury.

### Risk factors for preoperative brain injury

There were no clinical variables that were associated with increased risk of any cerebral lesion (online supplementary table 1), WMI (online supplementary table 2), AIS (online supplementary table 3) or cerebellar haemorrhage (online supplementary table 4). However, due to the small subgroup sample sizes involved, potentially quite large differences cannot be discounted from this study alone. There were no differences in GA at birth between infants with and without any cerebral lesion (p=0.45), WMI (p=0.32), AIS (p=0.51) or cerebellar haemorrhage (p=0.63). There was no difference between those with and without WMI for GA at scan (39.0 vs 39.6 weeks; p=0.14), or postnatal age in days (4 vs 6; p=0.07). There were no differences in the proportion of cases with or without any cerebral injury in infants with abnormal mixing (eg, TGA; n=34), left-sided lesions (eg, HLHS, CoA; n=14) and right-sided lesions (eg, TOF, pulmonary atresia; n=22). The only arterial infarcts were in infants with TGA, all of which followed balloon atrial septostomy (BAS). Those with TGA who underwent septostomy experienced stroke in 23% of cases (n=3/13, 95% CI 8% to 50%) compared with 0% of cases in those who did not undergo septostomy (n=0/15, 95% CI 0% to 20%), a difference of 23% (95% CI 2% to 50%; online supplementary table 5). AIS occurred in 33% (n=2/6, 95% CI 10% to 70%) of infants with septostomy performed via the umbilical vein, compared with 14% (n=1/7, 95% CI 3% to 51%) via the femoral vein (online supplementary table 2). Subdural haemorrhage occurred more frequently with induction of labour (49% vs 10%; p<0.001), normal vaginal delivery (52% vs 21%; p=0.01), ventouse delivery (83% vs 28%; p=0.013) and later GA at birth (p=0.005). Emergency caesarean was associated with reduced risk of subdural (5% vs 43%; p=0.003), with a similar trend observed in elective caesarean (8% vs 38%; p=0.09). Given the lack of clinical variables strongly associated with injury, we did not perform further logistic regression analysis to quantify joint associations.

Genetic testing was performed as part of routine clinical care in 83% of infants (n=58). Microarray was normal in 88% (51/58), with benign copy variant in 7% (4/58) and 22q11 deletion in 5% (3/58). Of those with 22q11, cerebellar haemorrhage was noted in 2/3, cerebellar vermis rotation in 1/3 and WMI in 1/3.

### Quantitative white matter lesion maps

**Figure 2:**
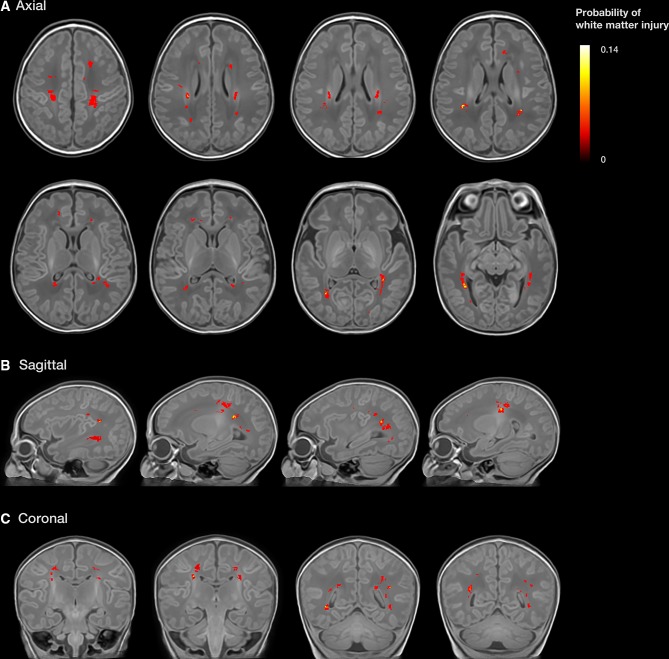
White matter injury probability map (n=22 included), superimposed onto a T1-weighted group template. WMI is demonstrated throughout the white matter including the frontal white matter, optic radiations, centrum semiovale and corona radiata. A three-dimensional representation of this figure is available in online supplementary video 1.

Quantitative maps of WMI were generated from 22 cases (1 infant excluded due to slight motion). WMI was distributed widely throughout the brain, involving the frontal white matter, optic radiations, centrum semiovale and corona radiata (figure 2). White matter lesions exhibited both restricted (48%) and normal (52%) signal on ADC maps, with no significant difference in days of age at scan between groups (p=0.35). There were no cases of haemorrhagic WMI identified using SWI. White matter lesion volume was not statistically associated with any clinical variable, which persisted after removing two outliers with large WMI burdens (CoA n=1, TGA n=1). A histogram of the distribution of white matter volume is presented in online supplementary figure 2.

10.1136/archdischild-2018-314822.supp2 Supplementary data

## Discussion

To our knowledge, this is the first prospective observational neuroimaging study in the UK of infants with CHD prior to surgery. We found cerebral lesions in 39% (95% CI 28% to  50%) of cases. WMI was the predominant lesion type, with few cases of AIS. WMI occurred at a rate over three times higher than in healthy term infants scanned contemporaneously at our institution using the same scanner and MRI acquisition protocols,^35^ with a widespread distribution including frontal white matter, optic radiations and corona radiata.

Preoperative WMI in infants with CHD has been reported in a number of studies previously.^36–44^ However, comparisons between cohorts are complicated by the heterogeneity of CHD studies, and homogenous cohorts with individual diagnoses are required to truly understand the risks for injury. In a recent study examining exclusively infants with HLHS preoperatively, WMI was observed in 11/57 (19%) of infants.^44^ Andropoulos *et al* reported preoperative WMI, infarction or haemorrhage in 28% of a cohort of 69 patients with both single and two ventricle pathology, and observed that there was no significant difference in preoperative WMI between single ventricle and 2 ventricle patients (13% vs 19%, respectively).^45^ The clinical consequence of WMI observed prior to surgery in infants with CHD is not well understood but appears to be related to the severity and location of the lesions. Moderate to severe WMI is associated with reduced cognitive scores at 2 years and lower full scale IQ at 6 years compared with CHD infants with no to mild WMI.^46^

The rate of WMI in infants with TGA in this study is consistent with other cohorts, which range from 14% to 38%.^25 36–38 42 43 47–49^ The incidence of arterial infarcts in infants with TGA has been reported between 5% and 29%,^7 25 36 43 49–51^ with our cohort at the lower end of this range. The relatively high prevalence of WMI in our cohort as a whole may be explained, at least in part, by a scan resolution that is higher than many comparable studies, potentially allowing smaller lesions to be discerned. Of note, most WMI in our cohort was mild or moderate, with only one infant having severe WMI. However, this would not explain our low incidence of AIS. Different local definitions of stroke, focal stroke, WMI, periventricular leucomalacia and punctate WMI may be responsible, and accurate comparisons are difficult without a consistent approach across institutions.

We found no cases of venous sinus thrombosis. The placement of central venous catheters in the internal jugular vein has been associated with increased risk of venous sinus thrombosis.^52^ In contrast to that study,^52^ our neonatal unit does not place subclavian or internal jugular vein catheters preoperatively, preferring instead umbilical venous catheters or peripherally inserted long lines. The absence of venous sinus thromboses in our cohort supports the view that internal jugular vein lines should be avoided in this population. We hypothesise that similar differences in clinical practice may reveal other important potential modifiable factors.

On evaluation of clinical parameters associated with cerebral lesions, we did not find clinical variables that were associated with an increased risk of WMI, AIS or cerebellar haemorrhage. Comparison of clinical variables with published cohorts is limited by detail available from previous studies. In our cohort, almost all infants were inborn at a tertiary cardiac centre and prenatally diagnosed. The true prenatal diagnosis rate in the UK is not currently known as there is no central registry. Around 50% of infants undergoing surgery in infancy in the UK have been diagnosed before birth, but this figure does not include terminations of pregnancy or stillbirth.^53^ Our region is known to have detection rates above the national average. Infants with prenatal diagnoses of CHD are known to have significantly less preoperative brain injury, thought to be due to improved cardiovascular stability following delivery,^54^ and may be an important factor to explain differences in incidences of brain injury between cohorts.

Subdural haemorrhage is common during term delivery, with an overall incidence of 8%–15%,^35 55^ and up to 28% in complicated instrumental deliveries.^55^ In our cohort, subdural haemorrhage occurred 5 times more frequently than reported in healthy normal vaginal delivery, and 10 times more frequently in ventouse delivery.^55^ At our institution, the timing of delivery is individualised, taking account of obstetric factors, where the parents live and potential requirement for early intervention. These factors impact on the mode of delivery and its timing. Induction was associated with a rate of subdural haemorrhage almost five times higher than in spontaneous onset of labour, which may partly be related to instrumental delivery being used over three times more frequently in this group. This association between induction and instrumental delivery contrasts with studies of healthy infants,^56^ and may be explained by a lower threshold for instrumental intervention in labours complicated by CHD, or potentially the use of induction at earlier GAs.

The timing of preoperative injury in CHD remains uncertain. Fetal MRI studies have not yet reported arterial strokes in utero, and few studies have identified white matter abnormalities prenatally.^16 57^ However, perinatal and postnatal injury is likely preceded by a period of abnormal brain development in utero, with reduced cerebral substrate^58^ and oxygen delivery,^59 60^ altered metabolism,^16^ and a derailed trajectory of fetal brain development in the third trimester.^15 16^ Following birth, there are continued metabolic disturbances^36 61^ and alterations in cerebral oxygen delivery.^62 63^ This chronic impairment may increase susceptibility to ischaemic injury around the time of delivery, a timeline that is supported by the finding that half of our cases exhibited WMI with restricted diffusion. If ischaemic, this would suggest an acute insult, although other aetiologies may explain restricted diffusion, including clusters of activated microglia resulting in increased cellularity.^64 65^

WMI was distributed throughout the white matter, in contrast to preterm infants where WMI is predominantly observed in the centrum semiovale and corticospinal tracts.^66 67^ While inflammatory and hypoxic–ischaemic injury to susceptible premyelinating oligodendrocytes^68^ may be responsible in both groups, it is possible that spatial differences in lesion distribution reflect developmental differences in the regional vulnerability of premyelinating oligodendrocytes between preterm infants and term infants with CHD. This vulnerability may be compounded by ischaemic vulnerability due to periventricular vascular anatomy, or by vascular congestion in the path of the medullary veins, leading to small venous infarcts, which may be responsible for the imaging appearances in the two infants with larger WMI.

Interestingly, all arterial infarcts in our study occurred following BAS. However, sample sizes were too small to form strong conclusions, and clinically important differences cannot be discounted from this study alone. Septostomy has been associated with an increased risk of cerebral infarction in some published studies,^7 69^ but not in others.^25 37 49^ All three arterial infarcts in our group were in the left middle cerebral artery territory, consistent with previous findings.^25 50^ Prediction of hypoxemia by prenatal echocardiography has proved difficult,^70^ and need for septostomy is generally assessed postnatally by the clinical team. It is therefore plausible that infants requiring septostomy will have experienced the greatest burden of hypoxia and cardiovascular instability after birth, and are most at risk of cerebral injury. The use of the umbilical vein has been implicated in displacement of pre-existing thrombus from the ductus venosus or hepatic vein during septostomy, increasing the risk of arterial embolic infarction.^71^ In this study, the umbilical route was associated with a greater proportion of AIS compared with femoral, although sample sizes were small. Previous studies showed no clear difference between the use of the femoral or umbilical vein.^7 69^

There were limitations to our study. The heterogeneous nature of the cohort and relatively small subgroup sample sizes hampered our ability to compare risk factors across groups. While this study adds a valuable new UK cohort to the literature, in isolation it does not advance greatly estimates of WMI prevalence from previous estimates. All infants were from a single centre, and almost all infants had been diagnosed antenatally and were inborn. Comparison of our results to other cohorts was complicated by the heterogeneity of CHD studies, the variable detail of clinical parameters in comparable studies and variability in radiological definitions across sites. Homogenous cohorts with individual diagnoses are required to truly understand the risks for injury.

## Conclusion

Cerebral lesions in newborns with CHD prior to surgery are common.
