# Supplementary material for: Neuroimaging findings in newborns with congenital heart disease prior to surgery: an observational study
Source: Arch Dis Child. 2019 Jun 26;104(11):1042–8. doi: 10.1136/archdischild-2018-314822 (PMC6801127; doi:10.1136/archdischild-2018-314822)

Supplementary Material

Neuroimaging findings in newborns with congenital heart disease prior to surgery: an observational study

Supplementary Table 1 Association of any lesion with clinical variables. "Brain lesion" includes arterial infarcts, punctate lesions and cerebellar haemorrhage (there were no cases of intraventricular haemorrhage, and subdural haemorrhage was not included). Data are expressed as % (n/total) or median (IQR), unless otherwise specified. *denotes normally-distributed variables, summarised by mean and standard deviation (SD). Comparison of categorical variables was performed using Fisher's exact tests with confidence intervals for the magnitude of the difference calculated using a two-sample test for equality of proportions. Normally distributed variables were compared using the Student's t-test. Non-normal continuous and ordinal variables were compared using the Mann-Whitney U test, with confidence intervals calculated using Hodges-Lehman estimation.

Variable	No lesion	Brain lesion	Difference (95% CI)	P value
Sex				
Female	46.5% (20/43)	48.1% (13/27)	1.6 (-21.1 - 24.3)	1.00
Male	53.5% (23/43)	51.9% (14/27)	-1.6 (-24.3 - 21.1)	1.00
Delivery method				0.87
Normal vaginal delivery	34.9% (15/43)	44.4% (12/27)	9.6 (-13.0 - 31.7)	0.46
Forceps vaginal delivery	9.3% (4/43)	7.4% (2/27)	-1.9 (-15.3 - 15.0)	1.00
Ventouse vaginal delivery	7.0% (3/43)	11.1% (3/27)	4.1 (-9.6 - 21.7)	0.67
Emergency caesarean	32.6% (14/43)	18.5% (5/27)	-14.0 (-32.2 - 7.8)	0.27
Elective caesarean	16.3% (7/43)	18.5% (5/27)	2.2 (-14.9 - 22.2)	1.00
Induction of labour	58.1% (25/43)	59.3% (16/27)	1.1 (-21.8 - 23.1)	1.00
Prenatal diagnosis	97.7% (42/43)	96.3% (26/27)	-1.4 (-16.1 - 8.8)	1.00
Outborn	2.3% (1/43)	3.7% (1/27)	1.4 (-8.8 - 16.1)	1.00
Gestational age at birth (weeks)	38.3 (37.4-38.7)	38.4 (37.6-38.9)	0.14 (-0.29, 0.71)	0.45
Gestational age at preoperative MRI (weeks)	39.0 (38.3-39.6)	39.3 (38.4-39.9)	0.28 (-0.29, 0.85)	0.35
Age at scan (days)	5 (2-7)	6 (3-9)	1.0 (-1.0, 3.0)	0.15
Birth weight (kg) (mean, SD)*	2.95 (0.61)	2.94 (0.52)	0.069 (-0.22, 0.36)	0.62
Head circumference (cm)	33.0 (32.3-34.5)	33.5 (32.0-34.5)	0.0 (-1.0, 1.0)	0.96
Apgar score				
1 min	9 (8-9)	9 (7-9)	0 (0, 0)	0.33
5 min	9 (9-10)	9 (9-10)	0 (0, 1)	0.42
Prostaglandin E2	37.2% (16/43)	55.6% (15/27)	18.3 (-5.2 - 39.5)	0.15
Cardiac arrest	0% (0/43)	0% (0/27)	0 (-8.2, 12.5)	1.00
Days mechanical ventilation	0 (0-0)	0 (0-2)	0 (0, 0)	0.029
Balloon atrial septostomy	14.0% (6/43)	25.9% (7/27)	12.0 (-6.5 - 32.1)	0.23
Femoral	7.0% (3/43)	14.8% (4/27)	7.8 (-6.8 - 26.1)	0.42
Umbilical	7.0% (3/43)	11.1% (3/27)	4.1 (-9.6 - 21.7)	0.67
Heart lesion				
Transposition of the great arteries (TGA)	41.9% (18/43)	37.0% (10/27)	-4.8 (-26.3 - 18.3)	0.80
Tetralogy of Fallot (TOF)	23.3% (10/43)	11.1% (3/27)	-12.1 (-28.4 - 7.6)	0.34
Coarctation of aorta (CoA)	9.3% (4/43)	25.9% (7/27)	16.6 (-1.1 - 36.2)	0.09
Pulmonary atresia	4.7% (2/43)	11.1% (3/27)	6.5 (-6.6 - 23.7)	0.37
Hypoplastic left heart syndrome (HLHS)	7.0% (3/43)	3.7% (1/27)	-3.3 (-15.3 - 12.0)	1.00
Pulmonary stenosis	2.3% (1/43)	7.4% (2/27)	5.1 (-6.0 - 21.2)	0.56
Truncus arteriosus	4.7% (2/43)	3.7% (1/27)	-0.9 (-12.2 - 14.0)	1.00
Tricuspid atresia	4.7% (2/43)	0.0% (0/27)	-4.7 (-15.5 - 8.3)	0.52
Large VSD	2.3% (1/43)	0.0% (0/27)	-2.3 (-12.1 - 10.3)	1.00

Supplementary Table 2 White matter injury association with clinical variables. Data are expressed as % (n/total) or median (IQR), unless otherwise specified. *denotes normally-distributed variables, summarised

by mean and standard deviation (SD). Comparison of categorical variables was performed using Fisher's exact tests with confidence intervals for the magnitude of the difference calculated using a two-sample test for equality of proportions. Normally distributed variables were compared using the Student's t-test. Non-normal continuous and ordinal variables were compared using the Mann-Whitney U test, with confidence intervals calculated using Hodges-Lehman estimation.

Variable	No WMI	WMI	Difference (95% CI)	P value
Sex				
Female	44.7% (21/47)	52.2% (12/23)	7.5 (-16.3 - 30.3)	0.62
Male	55.3% (26/47)	47.8% (11/23)	-7.5 (-30.3 - 16.3)	0.62
Delivery method				0.87
Normal vaginal delivery	38.3% (18/47)	39.1% (9/23)	0.8 (-21.3 - 24.5)	1.00
Forceps vaginal delivery	8.5% (4/47)	8.7% (2/23)	0.2 (-12.8 - 19.0)	1.00
Ventouse vaginal delivery	6.4% (3/47)	13.0% (3/23)	6.7 (-7.1 - 26.2)	0.39
Emergency caesarean	31.9% (15/47)	17.4% (4/23)	-14.5 (-32.2 - 8.3)	0.26
Elective caesarean	14.9% (7/47)	21.7% (5/23)	6.8 (-10.7 - 28.4)	0.51
Induction of labour	61.7% (29/47)	52.2% (12/23)	-9.5 (-32.5 - 13.9)	0.61
Prenatal diagnosis	97.9% (46/47)	95.7% (22/23)	-2.2 (-19.0 - 7.5)	1.00
Outborn	2.1% (1/47)	4.3% (1/23)	2.2 (-7.5 - 19.0)	1.00
Gestational age at birth (weeks)	38.3 (37.4-38.7)	38.4 (38.1-39.0)	0.28 (-0.28, 0.85)	0.32
Gestational age at preoperative MRI (weeks)	39.0 (38.3-39.6)	39.6 (38.4-40.0)	0.43 (-0.14, 1.0)	0.14
Age at scan (days)	4 (2-7)	6 (3-9)	2.0 (0.0, 4.0)	0.066
Birth weight (kg) (mean, 95% CI)*	2.90 (2.75 – 3.18)	2.96 (2.75 – 3.18)	0.055 (-0.24, 0.35)	0.71
Head circumference (cm)	33.5 (32.3-34.5)	33.5 (32.0-34.5)	-0.1 (-1.1, 0.8)	0.33
Apgar score				
1 min	9 (8-9)	9 (7-9)	0 (-1, 0)	0.20
5 min	9 (9-10)	9 (9-10)	0 (-1, 0)	0.57
Prostaglandin E2	40.4% (19/47)	52.2% (12/23)	11.7 (-12.2 - 34.3)	0.44
Cardiac arrest	0% (0/47)	0% (0/23)	0 (-7.6, 14.3)	1.00
Days mechanical ventilation	0 (0-0)	0 (0-2)	0 (-1, 0)	0.004
Balloon atrial septostomy	12.8% (6/47)	30.4% (7/23)	17.7 (-1.7 - 39.2)	0.10
Femoral	6.4% (3/47)	17.4% (4/23)	11.0 (-4.0 - 31.2)	0.21
Umbilical	6.4% (3/47)	13.0% (3/23)	6.7 (-7.1 - 26.2)	0.39
Heart lesion				
TGA	40.4% (19/47)	39.1% (9/23)	-1.3 (-23.4 - 22.5)	1.0
Tetralogy of Fallot	23.4% (11/47)	8.7% (2/23)	-14.7 (-29.9 - 5.9)	0.20
Coarctation of aorta	12.8% (6/47)	21.7% (5/23)	9.0 (-8.3 - 30.2)	0.49
HLHS	4.3% (2/47)	13.0% (3/23)	8.8 (-4.3 - 28.1)	1.0
Pulmonary atresia	6.4% (3/47)	4.3% (1/23)	-2.0 (-13.4 - 15.1)	0.32
Pulmonary stenosis	2.1% (1/47)	8.7% (2/23)	6.6 (-4.4 - 24.8)	0.25
Truncus arteriosus	4.3% (2/47)	4.3% (1/23)	0.1 (-10.5 - 17.0)	1.0
Tricuspid atresia	4.3% (2/47)	0.0% (0/23)	-4.3 (-14.2 - 10.4)	1.0
Large VSD	2.1% (1/47)	0.0% (0/23)	-2.1 (-11.1 - 12.3)	1.0

Supplementary Table 3 Association of arterial infarcts with clinical variables. Given infarcts only occurred in those with TGA, this table is restricted to the TGA subgroup. Data are expressed as % (n/total) or median (IQR), unless otherwise specified. *denotes normally-distributed variables, summarised by mean and standard deviation (SD). Given the small event size (n=3), P values have not been included in this table. Comparison of categorical variables was performed using Fisher's exact tests with confidence intervals for the magnitude of the difference calculated using a two-sample test for equality of proportions. Normally distributed variables were compared using the Student's t-test. Non-normal continuous and ordinal variables were compared using the Mann-Whitney U test, with confidence intervals calculated using Hodges-Lehman estimation.

Variable	No arterial infarct	Arterial infarct	Difference (95% CI)
Sex			
Female	56.0% (14/25)	0.0% (0/3)	-56.0 (-73.3 - 3.3)
Male	44.0% (11/25)	100.0% (3/3)	56.0 (-3.3 - 73.3)
Delivery method			
Normal vaginal delivery	40.0% (10/25)	33.3% (1/3)	-6.7 (-40.0 - 42.1)
Forceps vaginal delivery	16.0% (4/25)	0.0% (0/3)	-16.0 (-34.7 - 41.0)
Ventouse vaginal delivery	12.0% (3/25)	0.0% (0/3)	-12.0 (-30.0 - 44.7)
Emergency caesarean	16.0% (4/25)	33.3% (1/3)	17.3 (-15.6 - 64.2)
Elective caesarean	16.0% (4/25)	33.3% (1/3)	17.3 (-15.6 - 64.2)
Induction of labour	60.0% (15/25)	33.3% (1/3)	-26.7 (-58.5 - 23.1)
Prenatal diagnosis	96.0% (24/25)	100.0% (3/3)	4.0 (-52.2 - 19.5)
Outborn	4.0% (1/25)	0.0% (0/3)	-4.0 (-19.5 - 52.2)
Gestational age at birth (weeks)	38.5 (38.1 – 38.7)	38.1 (37.0 – 38.6)	-0.43 (-.157, 0.57)
Gestational age at preoperative MRI (weeks)	39.3 (38.6 – 39.6)	39.7 (37.0 – 39.9)	-0.29 (-2.29, 1.29)
Age at scan (days)	6 (4 – 7)	8 (0 – 12)	2 (-6, 7)
Birth weight (kg) *	3.03 (0.51)	2.84 (0.34)	-0.18 (-0.81, 0.44)
Head circumference (cm) *	33.3 (2.16)	32.2 (1.53)	-1.14 (-3.81, 1.52)
Apgar score			
1 min	9 (7 – 9)	6 (6 – 6)	-3 (-3, 0)
5 min	9 (8 – 9)	8 (6 – 8)	-1 (-3, 0)
Cord Arterial pH *	7.29 (0.073)	7.17 (0.19)	-0.13 (-0.28, 0.024)
Prostaglandin E2	48.0% (12/25)	33.3% (1/3)	-14.7 (-47.5 - 34.6)
Cardiac arrest	0% (0/25)	0% (0/3)	0 (-13, 56)
Days mechanical ventilation	0 (0 – 1)	2 (2 – 7)	2 (1, 7)
Balloon atrial septostomy	40.0% (10/25)	100.0% (3/3)	60.0 (0.6 - 76.6)
Femoral	24.0% (6/25)	33.3% (1/3)	9.3 (-24.1 - 56.9)
Umbilical	16.0% (4/25)	66.7% (2/3)	50.7 (1.1 - 79.5)

Supplementary Table 4 Association of cerebellar haemorrhage with clinical variables. Data are expressed as % (n/total) or median (IQR), unless otherwise specified. *denotes normally-distributed variables, summarised by mean and standard deviation (SD). Comparison of categorical variables was performed using Fisher's exact tests with confidence intervals for the magnitude of the difference calculated using a two-sample test for equality of proportions. Normally distributed variables were compared using the Student's t-test. Non-normal continuous and ordinal variables were compared using the Mann-Whitney U test, with confidence intervals calculated using Hodges-Lehman estimation.

Variable	No cerebellar haemorrhage	Cerebellar haemorrhage	Difference (95% CI)	P value
Sex				
Female	48.4% (31/64)	33.3% (2/6)	-15.1 (-41.6 - 23.4)	0.68
Male	51.6% (33/64)	66.7% (4/6)	15.1 (-23.4 - 41.6)	0.68
Delivery method				0.38
Normal vaginal delivery	35.9% (23/64)	66.7% (4/6)	30.7 (-7.9 - 56.7)	0.20
Forceps vaginal delivery	9.4% (6/64)	0.0% (0/6)	-9.4 (-19.0 - 30.0)	1.00
Ventouse vaginal delivery	7.8% (5/64)	16.7% (1/6)	8.9 (-7.6 - 48.8)	0.43
Emergency caesarean	28.1% (18/64)	16.7% (1/6)	-11.5 (-29.6 - 29.4)	1.00
Elective caesarean	18.8% (12/64)	0.0% (0/6)	-18.8 (-30.0 - 21.0)	0.58
Induction of labour	54.7% (35/64)	100.0% (6/6)	45.3 (4.6 - 57.4)	0.04
Prenatal diagnosis	96.9% (62/64)	100.0% (6/6)	3.1 (-36.0 - 10.7)	1.00
Outborn	3.1% (2/64)	0.0% (0/6)	-3.1 (-10.7 - 36.0)	1.00
Gestational age at birth (weeks)	38.3 (37.4 – 38.7)	38.6 (37.6 – 38.9)	0.15 (-1.00, 0.85)	0.63
Gestational age at preoperative MRI (weeks)	39.0 (38.4 – 39.7)	39.1 (38.6 – 39.7)	0.0 (-1.14, 1.00)	0.98
Age at scan (days)	5 (2 – 7)	5 (4 – 7)	0 (-3, 2)	0.93
Birth weight (kg) *	2.91 (0.58)	3.03 (0.64)	0.12 (-0.37, 0.62)	0.24
Head circumference (cm) *	33.2 (1.94)	34.1 (2.25)	0.81 (-0.87, 2.50)	0.34
Apgar score				
1 min	9 (7 – 9)	9 (8 – 9)	0 (0, 1)	0.74
5 min	9 (9 – 10)	9 (8 – 9)	0 (-1, 0)	0.31
Prostaglandin E2	42.2% (27/64)	66.7% (4/6)	24.5 (-14.2 - 50.7)	0.40
Cardiac arrest	0% (0/64)	0% (0/6)	0 (-5.7, 39)	1.00
Days mechanical ventilation	0 (0 – 0.5)	0 (0 – 2)	0 (0, 0)	0.75
Balloon atrial septostomy	18.8% (12/64)	16.7% (1/6)	-2.1 (-19.8 - 38.3)	1.00
Femoral	9.4% (6/64)	16.7% (1/6)	7.3 (-9.4 - 47.3)	0.48
Umbilical	9.4% (6/64)	0.0% (0/6)	-9.4 (-19.0 - 30.0)	1.00
Heart lesion				
TGA	40.6% (26/64)	33.3% (2/6)	-7.3 (-33.9 - 31.0)	1.00
Tetralogy of Fallot	18.8% (12/64)	16.7% (1/6)	-2.1 (-19.8 - 38.3)	1.00
Coarctation of aorta	14.1% (9/64)	33.3% (2/6)	19.3 (-6.6 - 56.5)	0.24
Pulmonary atresia	6.2% (4/64)	16.7% (1/6)	10.4 (-5.8 - 50.3)	0.37
HLHS	6.2% (4/64)	0.0% (0/6)	-6.3 (-15.0 - 33.0)	1.00
Pulmonary stenosis	4.7% (3/64)	0.0% (0/6)	-4.7 (-12.9 - 34.5)	1.00
Truncus arteriosus	4.7% (3/64)	0.0% (0/6)	-4.7 (-12.9 - 34.5)	1.00
Tricuspid atresia	3.1% (2/64)	0.0% (0/6)	-3.1 (-10.7 - 36.0)	1.00
Large VSD	1.6% (1/64)	0.0% (0/6)	-1.6 (-8.3 - 37.5)	1.00

Supplementary Table 5 Contingency table to show risk of arterial stroke in infants with TGA following balloon atrial septostomy

	**Stroke**	**No stroke**	**Total**
**Balloon atrial septostomy**	3	10	13
**No balloon atrial septostomy**	0	15	15
**Total**	3	25	28

Supplementary Table 6 White matter injury association with clinical variables in infants with abnormal mixing. Data are expressed as % (n/total) or median (IQR), unless otherwise specified. *denotes normally-distributed variables, summarised by mean and standard deviation (SD). Comparison of categorical variables was performed using Fisher's exact tests with confidence intervals for the magnitude of the difference calculated using a two-sample test for equality of proportions. Normally distributed variables were compared using the Student's t-test. Non-normal continuous and ordinal variables were compared using the Mann-Whitney U test, with confidence intervals calculated using Hodges-Lehman estimation.

Variable	No WMI	WMI	Difference (95% CI)	P value
Sex				
Female	52.2% (12/23)	36.4% (4/11)	-15.8 (-44.0 - 18.4)	0.48
Male	47.8% (11/23)	63.6% (7/11)	15.8 (-18.4 - 44.0)	0.48
Delivery method				
Normal vaginal delivery	34.8% (8/23)	45.5% (5/11)	10.7 (-20.9 - 41.6)	0.71
Forceps vaginal delivery	17.4% (4/23)	0.0% (0/11)	-17.4 (-37.1 - 10.5)	0.28
Ventouse vaginal delivery	8.7% (2/23)	9.1% (1/11)	0.4 (-19.2 - 29.7)	1.00
Emergency caesarean	21.7% (5/23)	18.2% (2/11)	-3.6 (-27.6 - 28.3)	1.00
Elective caesarean	17.4% (4/23)	27.3% (3/11)	9.9 (-16.5 - 41.0)	0.66
Induction of labour	60.9% (14/23)	45.5% (5/11)	-15.4 (-45.0 - 17.9)	0.48
Prenatal diagnosis	100.0% (23/23)	90.9% (10/11)	-9.1 (-37.7 - 7.1)	0.32
Outborn	0.0% (0/23)	9.1% (1/11)	9.1 (-7.1 - 37.7)	0.32
Gestational age at birth (weeks)	38.3 (37.6 – 38.7)	38.4 (38.1 – 38.7)	0.14 (-0.42, 1)	0.56
Gestational age at preoperative MRI (weeks)	39.0 (38.4 – 39.4)	39.3 (38.4 – 39.9)	0.42 (-0.43, 1.42)	0.29
Age at scan (days)	6 (4 – 7)	7 (1 – 11)	2 (-2, 5)	0.33
Birth weight (kg) (mean, 95% CI)*	2.92 (0.53)	2.91 (0.49)	-0.019 (-0.41, 0.37)	0.92
Head circumference (cm) *	33.4 (1.89)	32.8 (2.03)	-0.60 (-2.04, 0.85)	0.92
Apgar score				
1 min	9 (6 – 9)	9 (6 – 9)	0 (-1, 0)	0.89
5 min	9 (7 – 9)	9 (8 – 10)	0 (-1, 1)	0.56
Prostaglandin E2	34.8% (8/23)	54.5% (6/11)	19.8 (-13.7 - 48.7)	0.458
Cardiac arrest	0% (0/23)	0% (0/11)	0 (-14.3, 25.9)	1.00
Days mechanical ventilation	0 (0 – 0)	2 (0 – 3)	1 (0 – 2)	0.023
Balloon atrial septostomy	26.1% (6/23)	63.6% (7/11)	37.5 (2.7 - 62.7)	0.178
Femoral	13.0% (3/23)	36.4% (4/11)	23.3 (-5.2 - 52.8)	0.363
Umbilical	13.0% (3/23)	27.3% (3/11)	14.2 (-11.7 - 44.7)	0.06

Supplementary Figure 1 Histogram of age at first cardiac intervention or surgery (excluding balloon atrial septostomy).

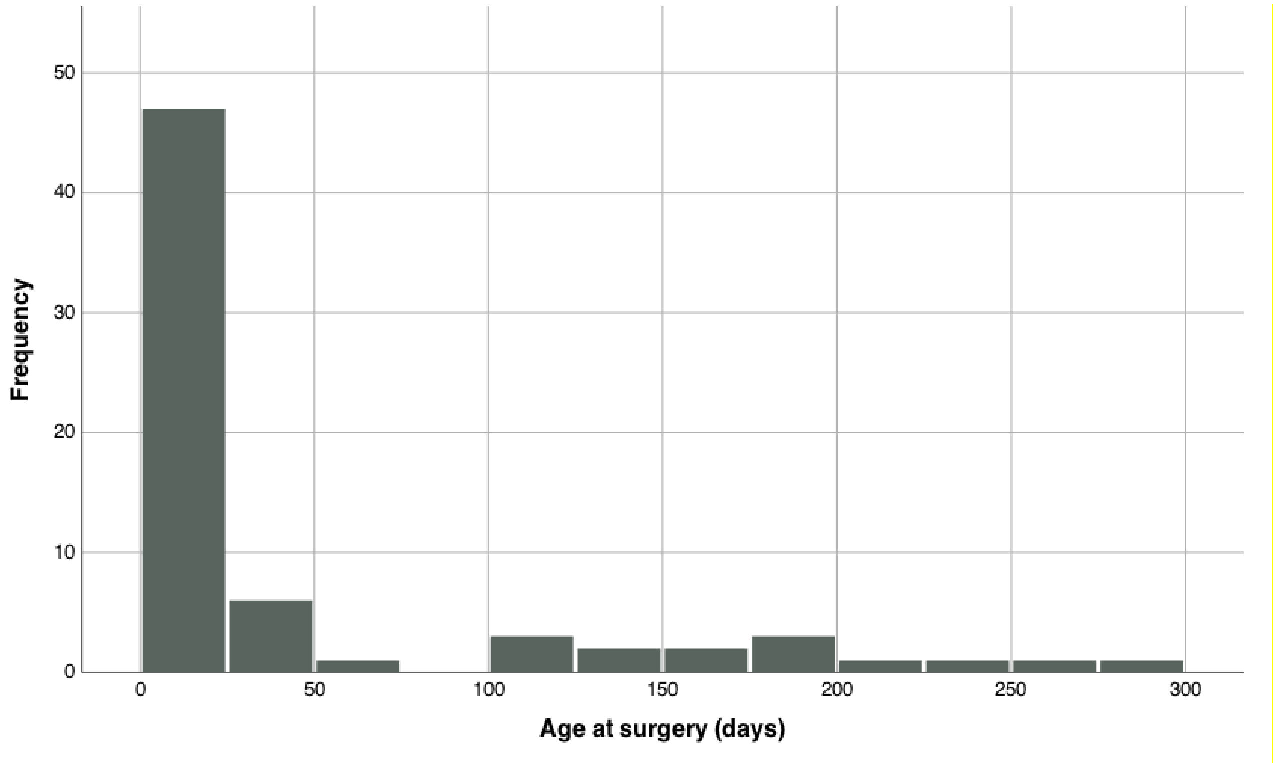

Supplementary Figure 2 Histogram of white matter injury volume, presented as a percentage of total brain volume. White matter injury volumes were manually segmented from T1-weighted imaging. For clarity of presentation, two outliers have been excluded: i) TGA - 6.5% and ii) CoA - 5.8%.

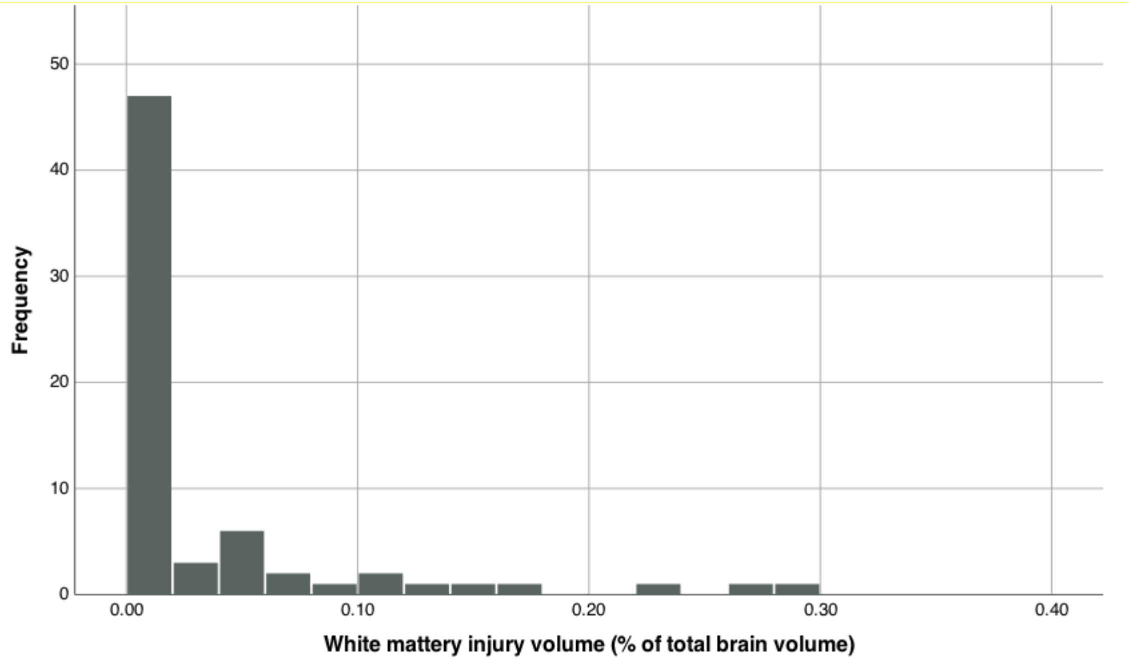

Supplement: Supplementary data [file archdischild-2018-314822supp001.pdf]
